# Stage-specific expression of protease genes in the apicomplexan parasite, *Eimeria tenella*

**DOI:** 10.1186/1471-2164-13-685

**Published:** 2012-12-07

**Authors:** Marilyn Katrib, Rowan J Ikin, Fabien Brossier, Michelle Robinson, Iveta Slapetova, Philippa A Sharman, Robert A Walker, Sabina I Belli, Fiona M Tomley, Nicholas C Smith

**Affiliations:** 1 Institute for the Biotechnology of Infectious Diseases, University of Technology, 2007, Sydney, Broadway, N.S.W., Australia; 2 Queensland Tropical Health Alliance Research Laboratory, Faculty of Medicine, Health and Molecular Sciences, James Cook University, Cairns Campus, McGregor Road, 4878, Smithfield, QLD, Australia; 3 INRA UR1282, Equipe Pathogenèse des Coccidioses, Infectiologie Animale et Santé Publique, 37380, Nouzilly, France; 4 Royal Veterinary College, Hawkshead Lane, North Mymms, AL9 7TA, Hatfield, Hertfordshire, UK

**Keywords:** *Eimeria*, Apicomplexa, Protease, Protease inhibitors, Gametocyte, Oocyst wall

## Abstract

**Background:**

Proteases regulate pathogenesis in apicomplexan parasites but investigations of proteases have been largely confined to the asexual stages of *Plasmodium falciparum* and *Toxoplasma gondii*. Thus, little is known about proteases in other Apicomplexa, particularly in the sexual stages. We screened the *Eimeria tenella* genome database for proteases, classified these into families and determined their stage specific expression.

**Results:**

Over forty protease genes were identified in the *E. tenella* genome. These were distributed across aspartic (three genes), cysteine (sixteen), metallo (fourteen) and serine (twelve) proteases. Expression of at least fifteen protease genes was upregulated in merozoites including homologs of genes known to be important in host cell invasion, remodelling and egress in *P. falciparum* and/or *T. gondii*. Thirteen protease genes were specifically expressed or upregulated in gametocytes; five of these were in two families of serine proteases (S1 and S8) that are over-represented in the coccidian parasites, *E. tenella* and *T. gondii,* distinctive within the Apicomplexa because of their hard-walled oocysts. Serine protease inhibitors prevented processing of EtGAM56, a protein from *E. tenella* gametocytes that gives rise to tyrosine-rich peptides that are incorporated into the oocyst wall.

**Conclusion:**

*Eimeria tenella* possesses a large number of protease genes. Expression of many of these genes is upregulated in asexual stages. However, expression of almost one-third of protease genes is upregulated in, or confined to gametocytes; some of these appear to be unique to the Coccidia and may play key roles in the formation of the oocyst wall, a defining feature of this group of parasites.

## Background

Proteases are essential regulators of pathogenesis in the Apicomplexa, a phylum that includes obligate, intracellular protozoan parasites of great human health (e.g., *Plasmodium* species, causing malaria, *Toxoplasma gondii,* causing toxoplasmosis, and *Cryptosporidium,* causing cryptosporidiosis) and agricultural and economic significance (e.g., *Neospora caninum,* the cause of foetal abortion in cattle, and *Eimeria* species, the causative agents of coccidiosis in poultry, cattle, sheep and rabbits). Extensive study of *Plasmodium* species and *T. gondii* has established that proteases help to coordinate and regulate the lifecycles of these parasites, playing key roles in host cell invasion, general catabolism, host cell remodelling and egress from host cells [1]. These processes are all associated with the asexual stages of apicomplexan parasites. By contrast, relatively little is known about what roles proteases may play in the sexual phase of the apicomplexan lifecycle though it is known that a subtilisin 2 is detected specifically in the gametocyte proteome [2] and expression of *falcipain 1* is upregulated in gametocytes [3] of *P. falciparum.* Moreover, it has been demonstrated that the cysteine protease inhibitor, E64d, or the targeted genetic disruption of *falcipain 1* can inhibit oocyst production in *P. falciparum*[3,4]. Likewise, the proteosome inhibitors, epoxomicin and thiostrepin, exhibit gametocytocidal activity [5,6].

In comparison to *P. falciparum* and *T. gondii,* proteases from *Eimeria* species have been studied far less intensively, despite the economic importance of this genus of parasites. Thus, homologs or orthologs of several classes of proteases found in *P. falciparum* and/or *T. gondii* have also been identified in *Eimeria* species including an aspartyl protease [7-10], an aminopeptidase [11], a rhomboid protease [12,13], a subtilisin 2-like protease [10,13,14], three cathepsin Cs [15], a cathepsin L [15] and an orthologue of toxopain, a cathepsin B cysteine protease [14,15]. As for *P. falciparum* and *T. gondii,* these proteases have been found in the asexual stages of *Eimeria* and are mostly predicted to play roles in host cell invasion, though expression of some of these enzymes is associated with the sporulation of the developing oocyst [11,13,15]. However, it is hypothesized that proteolytic processing of two proteins from the wall forming bodies of the macrogametocytes of *Eimeria* – GAM56 and GAM82 – is essential for the subsequent incorporation of tyrosine-rich peptides into the oocyst wall [16].

In this study, we screened the *E*. *tenella* genomic database for genes encoding proteases, classified these into clans and families and designed PCR probes for them. Using cDNA produced from *E*. *tenella* stage specific mRNA, we carried out semi-quantitative PCR to determine the stage specificity of expression of the protease genes, especially to identify protease mRNAs that were upregulated in gametocytes. In order to further resolve which of these may be involved in oocyst wall formation, we carried out a processing assay using gametocyte extracts of *E*. *tenella*, whereby a variety of specific protease inhibitors were tested for their ability to inhibit the processing of GAM56 into smaller, putative oocyst wall proteins.

## Results

### *Identification of potential protease genes in* Eimeria tenella

The genome of *E. tenella* (Houghton strain) was sequenced by the Parasite Genomics Group at the Wellcome Trust Sanger Institute and provided pre-publication for the current analysis. The Parasite Genomics Group plan to publish the annotated sequence in a peer-reviewed journal in the coming future. The *E. tenella* genome database (http://www.genedb.org/Homepage/Etenella) was explored to identify genes that were automatically predicted to code for aspartic, cysteine, metallo and serine proteases. Database mining revealed over 60 gene sequences whose predicted open reading frames were associated with potential peptidase activity. Manual annotation of the genes was performed by BLAST search of apicomplexan genome databases to identify phylogenetically closely related nucleotide sequences and by BLAST search of various protein databases to identify the most closely related, experimentally characterized homologs available (Table 1). Additionally, the predicted proteins were analysed for conserved motifs and domains to further validate protein function (Table 1). Each predicted protein was then assigned a five-tiered level of confidence for function using an Evidence Rating (ER) system (Table 1). The evidence rating system, described previously [17], allocates genes an overall score (ER1-5), indicating how compelling the bioinformatic and experimental evidence is for protein function. An ER1 rating signifies extremely reliable experimental data to support protein function in the particular species being investigated, in this case *Eimeria,* whereas ER5 indicates no experimental or bioinformatic evidence for gene function. Genes with an ER5 were eliminated from further investigation. After this validation process was performed, 45 putative protease genes remained and these could be classified into clans and families of aspartic, cysteine, metallo and serine proteases (Table 1), including: three aspartic proteases, all within family A1 in clan AA; 16 cysteine proteases, the vast majority (15) of which were in clan CA, five being cathepsins (family C1), one calpain (family C2), eight ubiquitinyl hydrolases (family C19) and one OTU protease (family C88), as well as a single clan CF pyroglutamyl peptidase (family C15); 14 metallo proteases, distributed over five clans (MA (6), ME (5), MF (1), MK (1) and MM (1)) and seven families (M1 (2), M41 (3), M48 (1), M16 (5), M17 (1), M22 (1) and M50 (1)); and 12 serine proteases in clan PA (three trypsin-like proteases in family S1), clan SB (six subtilisin-like proteases in family S8), clan SC (one prolyl endopeptidase in family S33), clan SK (one Clp protease in family S14) and clan ST (a rhomboid protease – rhomboid protease 1 – in family S54). Three additional rhomboid proteases were identified in the *E. tenella* genome database by using BLASTP to search the database using, as queries, homologs described in *T. gondii:* rhomboid protease 3 (ETH_00032220, Supercontig_69: 140161–141340; 4.0e52); rhomboid protease 4 (ETH_00009820, Supercontig_44: 17996–24858; 9.8e-164); and rhomboid protease 5 (ETH_00040480, contig NODE_916_length_3953_cov_17.775614: 53–3466; 7.2e-65). However, we were unable to confirm coding sequences or stage-specific expression for any of these three genes.

**Table 1:** **Protease genes identified in the *Eimeria tenella *** **genome database**

**Protease/Gene Identifier/Contig**	**Clan**	**Family**	**BLAST Apicomplexa (Database: nucleotide)**	**BLAST NCBI (Database: PDB, Swissprot, NR)**	**Family Domains (Pfam, MEROPS, InterProScan)**	**Evidence Rating**
**Aspartic Proteases**						
Eimepsin 1 ETH_00001725 on Supercontig_54	AA	A1	*Theileria annulata* strain Ankara genomic DNA chromosome 3 (E=2e-17)	**PDB:** Porcine Pepsin (E=4e-11)	partial	ER1
Eimepsin 2 ETH_00007420 on Supercontig_38	AA	A1	*Toxoplasma gondii* ME49 gcontig_1112359860822 (E=7e-36)	**NR:** aspartic protease 7 [*Toxoplasma gondii*] (E=3e-37)	none	ER3/4
Eimepsin 3 ETH_00008525 on Supercontig_8	AA	A1	*Plasmodium berghei* whole genome shotgun assembly, contig PB_RP2841 (E=1e-93)	**PDB:** Human pepsin (E=7e-56)	complete	ER2
**Cysteine Proteases**						
Cathepsin B ETH_00003570 on Supercontig_23	CA	C1	*Toxoplasma gondii* GAB2-2007-GAL-DOM2 contig00350 (E= 1e-12)	**PDB:** Human Recombinant Procathepsin B (E=2e-58)	Complete	ER2
Cathepsin L ETH_00033530 on NODE_2923_length_1315_cov_12.253232	CA	C1	*Toxoplasma gondii* ME49 gcontig_1112359872114 (E= 9e-43)	**PDB:***Toxoplasma gondii* Cathepsin L (Tgcpl) (E=1e-64)	Complete	ER2
Cathepsin C1 ETH_00019750 on Supercontig_2	CA	C1	*Toxoplasma gondii* ME49 gcontig_1112359873648 (E= 5e-46)	**PDB:** Porcine Cathepsin H (E=3e-11)	Partial	ER2/3
Cathepsin C2 ETH_0005000 on NODE_22022_length_2554_cov_8.124119	CA	C1	*Toxoplasma gondii* GT1 gcontig_1107000835548 (E=2e-11)	**PDB:** Human Dipeptidyl Peptidase I (Cathepsin C) (E=0.016)	Partial	ER3/4
Cathepsin C3 ETH_00001590, ETH_00001595 and ETH_00001600 on Supercontig_115	CA	C1	*Cryptosporidium hominis* strain TU502 chromosome 4 CHRO014106 (E=1e-34)	**PDB:** Cathepsin C *Rattus norvegicus* (E= 3e-13)	partial	ER2
Calpain ETH_00004075 on Supercontig_49	CA	C2	*Toxoplasma gondii* ME49 gcontig_1112359873650 (E=5e-96)	**PDB:** Human Calpain 8 (E= 1e-16)	Complete	ER2
Ubiquitinyl hydrolase 1 ETH_00012075 on Supercontig_122	CA	C19	*Cryptosporidium muris* RN66 gcontig_1106632353963 (E=2e-87)	**Swiss-Prot:** ubiquitin specific peptidase 39 [*Mus musculus*] (E=1e-116)	Complete	ER2
Ubiquitinyl hydrolase 2 ETH_00034675 on Supercontig_3	CA	C19	*Cryptosporidium muris* RN66 gcontig_1106632353937 (E=3e-35)	**Swiss-Prot:** ubiquitin specific peptidase 5 (isopeptidase T)[*Mus musculus*] (E=7e-81)	Partial	ER2
Ubiquitinyl hydrolase 3 ETH_00001555 on Supercontig_115	CA	C19	*Neospora caninum* Liverpool ubiquitin carboxyl-terminal hydrolase, related (NCLIV_041690) mRNA, partial cds (E=63e-94)	**PDB:** Ubp-Family Deubiquitinating Enzyme [human] (E= 5e-40)	Complete	ER2
Ubiquitinyl hydrolase 4 ETH_00007310 on Supercontig_39	CA	C19	*Cryptosporidium muris* RN66 gcontig_1106632353835 (E=1e-60)	**PDB:** Usp14, A Proteasome-Associated Deubiquitinating Enzyme (E= 5e-40)	Complete (disrupted)	ER2
Ubiquitinyl hydrolase 5 ETH_00003260 on Supercontig_106	CA	C19	*Plasmodium falciparum* VS/1 cont1.2577 (E=2e-49)	**PDB:** Human Ubiquitin Carboxyl-Terminal Hydrolase 8 (E=2e-37)	Partial	ER2
Ubiquitinyl hydrolase 6 ETH_00020635 on Supercontig_5	CA	C19	*Toxoplasma gondii* GT1 gcontig_1107000919460 (E=1e-12)	**Swiss-Prot:** Ubiquitin carboxyl-terminal hydrolase 26 *Arabidopsis thaliana *(E= 2e-12)	Partial	ER2/3
Ubiquitinyl hydrolase 7 ETH_00008925 on Supercontig_8	CA	C19	*Plasmodium vivax* SaI-1 ctg_6569 (E=1e-30)	**PDB:** Ubiquitin-Usp2 Complex [human] (E= 2e-15)	Partial	ER2
Ubiquitinyl hydrolase 8 ETH_00003260 on Supercontig_106	CA	C19	*Neospora caninum* Liverpool Ubiquitin carboxyl-terminal hydrolase, related (NCLIV_024510) mRNA, complete cds (E=8e-17)	**PDB:** Covalent Ubiquitin-Usp2 Complex [human] (E= 4e-10)	Partial	ER2/3
OTU protease no gene name on NODE_10106_length_3351_cov_7.612056	CA	C88	*Toxoplasma gondii* ME49 gcontig_1112359861240 (E=3e-9)	**PDB:** OTU [*Saccharomyces cerevisiae*] (E= 2e-11)	None	ER3/4
Pyroglutamyl peptidase ETH_00030160 on Supercontig_14	CF	C15	*Toxoplasma gondii* ME49 gcontig_1112359873116 (E=0.1)	**Swiss-Prot:** Pyrrolidone Carboxyl Peptidase (pyroglutamyl peptidase) *Chromobacterium violaceum* (E= 8e-5)	Partial	ER3
**Metallo Proteases**						
Aminopeptidase N 1 ETH_00013105 on Supercontig_9	MA	M1	*Neospora caninum* Liverpool complete genome, chromosome X (E=1e-24)	**PDB:** Aminopeptidase N From Human Pathogen *Neisseria meningitidis* (E=1e-144)	Partial	ER2
Aminopeptidase N 2 ETH_00015595 on Supercontig_153	MA	M1	*Babesia bovis* strain T2Bo chromosome 4 gcontig_1104837696308 (E=7e-57)	**PDB:** M1 Alanylaminopeptidase From Malaria (E= 4e-65)	Partial	ER2
ATP-dependant Zn protease 1 no gene name on NODE_975_length_1397_cov_15.574087	MA	M41	*Plasmodium falciparum* FCC-2/Hainan cont1.4384 (E=9e-31)	**PDB:** Ftsh Protease Domain [*Aquifex aeolicus*] (E=1e-21)	Complete	ER2
ATP-dependant Zn protease 2 ETH_00018435 on Supercontig_60	MA	M41	*Plasmodium falciparum* VS/1 cont1.4464 (E=2e-68)	**PDB:** Ftsh [*Escherichia coli*] (E=1e-65)	Complete	ER2
ATP-dependant Zn protease 3 ETH_00010985 on Supercontig_4	MA	M41	*Toxoplasma gondii* ME49 gcontig_1112359860098 (E=2e-54)	**PDB:** Human Paraplegin (FtsH endopeptidase family) (E=7e-40)	Partial	ER3
CaaX prenyl protease ETH_00017305 on Supercontig_76	MA	M48	*Babesia bovis* strain T2Bo chromosome 4 gcontig_1104837696380 (E=2e-77)	**Swiss-Prot:** CAAX prenyl protease 1 homolog [*Arabidopsis thaliana*] (E=2e-83)	Complete	ER2
Insulysin 1 ETH_00011835 on Supercontig_36	ME	M16	*Plasmodium vivax* SaI-1 ctg_7222 (E=5e-168)	**PDB:** Bovine Bc1 (Zn-dependant insulinase) (E=1e-101)	Complete	ER2
Insulysin 2 ETH_00032950 on Supercontig_103	ME	M16	*Babesia bovis* T2Bo chromosome 3 (E=1e-133)	**PDB:** Yeast Mitochondrial Processing Peptidase (E=4e-60)	Complete	ER2
Insulysin 3 ETH_00001730 on Supercontig_54	ME	M16	*Toxoplasma gondii* ME49 gcontig_1112359871056 (E=3e-90)	**PDB:** Human insulin degrading enzyme (Ide) (E=2e-46)	Complete	ER1/2
Insulysin 4 no gene name on Supercontig_901	ME	M16	*Toxoplasma gondii* VEG gcontig_1104442817478 (E=1e-22)	**PDB:** Human insulin degrading enzyme (Ide) (E=3e-17)	Partial	ER2/3
Insulysin 5 no gene name on NODE_2627_length_1769_cov_14.530243	ME	M16	-	**PDB:** Pitrilysin (M16 family) [*Escherichia coli* O157:H7] (E=7e-05)	None	ER4
Leucine aminopeptidase ETH_00012380 on Supercontig_27	MF	M17	*Toxoplasma gondii* ME49 cytosol aminopeptidase, mRNA (E=4e-54)	**PDB:***E. coli* Aminopeptidase A (Pepa) (E=1e-53)	Complete	ER2
O-sialoglycoprotease ETH_00020530 on Supercontig_5	MK	M22	*Toxoplasma gondii* ME49 glycoprotease family domain-containing protein, mRNA (E=1e-47)	**PDB:***Methanococcus jannaschii* Kae1-Bud32 Fusion Protein (Kae1: sialoglycoprotease homologue) (E=1e-51)	Complete (disrupted)	ER2
S2P-like protease ETH_00009130 on Supercontig_80	MM	M50	-	**NR:** peptidase, M50 family protein [*Toxoplasma gondii*] (E=4e-21)	Complete	ER3
**Serine Proteases**						
Trypsin 1 ETH_00028355 on Supercontig_45	PA	S1	*Babesia bovis* strain T2Bo chromosome 4 gcontig_1104837696308 (E=1e-56)	**Swiss-Prot:** Protease Do-like 10 [*Arabidopsis thaliana*] (E=2e-63)	Complete	ER2
Trypsin 2 ETH_00012215 on Supercontig_27	PA	S1	*Neospora caninum* Liverpool complete genome, chromosome IX (E=1e-21)	**Swiss-Prot:** Protease Do-like 9 [*Arabidopsis thaliana*] (E=1e-63)	Complete	ER2/3
Trypsin 3 ETH_00015245 on Supercontig_30	PA	S1	*Toxoplasma gondii* ME49 gcontig_1112359861240 (E=6e-58)	**Swiss-Prot:** Protease Do-like 2 [*Arabidopsis thaliana*] (E=2e-80)	Complete	ER2
Subtilisin 1 ETH_00009790 on Supercontig_570	SB	S8	*Cryptosporidium parvum* Iowa II chromosome 6 chr6.s2 (E=2e-22)	**Swiss-Prot:** Cell wall-associated protease [*Bacillus subtilis*] (E=8e-18)	Partial	ER2
Subtilisin 2 ETH_00025145 on Supercontig_1463	SB	S8	*Cryptosporidium muris* RN66 gcontig_1106632353939 (E=8e-18)	**Swiss-Prot:** Major intracellular serine protease [*Bacillus subtilis*] (E=4e-9)	Partial	ER1/2
Subtilisin 3 ETH_00011050 on Supercontig_4	SB	S8	*Cryptosporidium muris* RN66 gcontig_1106632353939 (E=1e-39)	**PDB:** Subtilisin [*Bacillus licheniformis*] (E=3e-28)	Complete	ER2
Subtilisin 4 ETH_00006825 on Supercontig_65	SB	S8	*Cryptosporidium parvum* Iowa II chromosome 6 chr6.s2 (E=3e-53)	**PDB:** Thermitase [*Thermoactinomyces vulgaris*] (E=3e-32)	Complete	ER2
Subtilisin 5 ETH_00011340 on Supercontig_4	SB	S8	*Toxoplasma gondii* ME49 gcontig_1112359859078 (E=3e-24)	**PDB:** Thermostable Serine Protease [*Bacillus sp*] (E=8e-7)	Partial	ER3/4
Subtilisin 6 ETH_00016890 on Supercontig_22	SB	S8	*Toxoplasma gondii* VEG gcontig_1104442818966 (E=6e-34)	**PDB:** Thermitase [*Thermoactinomyces vulgaris*] (E= 4e-11)	Partial	ER3/4
Prolyl endopeptidase ETH_00028960 on Supercontig_1	SC	S33	*Neospora caninum* Liverpool complete genome, chromosome V (E=2e-6)	**Swiss-Prot:** prolyl endopeptidase [*Mus musculus*] (E=4e-98)	Complete	ER2
Clp protease ETH_00030480 on Supercontig_126	SK	S14	-	**Swissprot:** ATP-dependent Clp protease proteolytic subunit [*Neisseria meningitidis*] (E= 3e-11)	Partial	ER3/4
Rhomboid protease ETH_00020020 on Supercontig_2	ST	S54	*Plasmodium falciparum* Santa Lucia cont1.4986, (E=1e-23)	**Swiss-Prot:** Rhomboid-like protease 1 [*Toxoplasma gondii*] (E=3e-47)	Complete	ER1/2

The *E. tenella* genome database (http://www.genedb.org/Homepage/Etenella) was searched for genes predicted to code for proteins with peptidase activity. All auto-annotated peptidase genes identified were manually curated by performing BLAST analysis against apicomplexan genome sequence databases and various protein databases [32] such as the protein data bank (PDB), Swiss-Prot and non-redundant (NR). In addition, signature protein motifs for the protein sequence of each gene were identified through Pfam (http://pfam.sanger.ac.uk/search; [33]), InterproScan (http://www.ebi.ac.uk/Tools/pfa/iprscan/) and the MEROPS databases (http://merops.sanger.ac.uk/; [34]). Further gene sequence manipulations, such as translation into amino acid sequences and ClustalW alignments, were performed using the DNASTAR Lasergene™ 9 Core Suite. Genes were assigned a five-tiered level of confidence for gene function using an Evidence Rating (ER) system giving an overall score of ER1-5, where ER1 indicates extremely reliable experimental data to support function and ER5 indicates no evidence for gene function [17].

### Stage-specific protease gene expression

**Figure 1:**
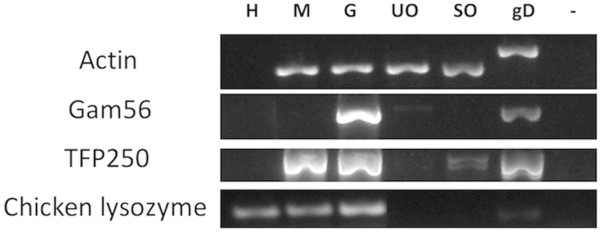
**RT-PCR assessment of purity of *Eimeria tenella *merozoites, gametocytes and oocysts.** Total RNA was extracted from *E. tenella* merozoite (M), gametocyte (G), unsporulated oocyst (UO) and sporulated oocyst (SO) preparations. cDNA was synthesized and stage-specific parasite genes (GAM56 for gametocytes; TFP250 for merozoites and sporozoites) was amplified by RT-PCR. PCR samples were run alongside chicken host cDNA (H), *E. tenella* genomic DNA (gD) and a negative control sample with no template (−) on a 1% agarose gel. The *E. tenella* β-actin gene product was used to standardize loading of parasite cDNA samples.

To assess the stage specific gene expression of putative proteases identified in the *E. tenella* database, different stages of the parasite lifecycle were isolated and total RNA purified. These stages included merozoites, 134 h gametocytes, unsporulated oocysts, sporulated oocysts as well as uninfected caeca control tissue. RT-PCR was performed and the stage-specific cDNA samples were subjected to control PCRs to determine purity (Figure 1). Purification of merozoite and gametocyte lifecycle stages inevitably results in co-purification of host tissue, hence, the *E. tenella* β-actin structural gene was amplified to optimize relative amounts of parasite starting material as described previously [18]. The *E. tenella* β-actin gene was amplified from each of the parasite lifecycle cDNA samples and quantification of bands visualized by agarose gel electrophoresis allowed the specific *E. tenella* cDNA to be standardized to each other accordingly. The *E. tenella gam56* gene product, which is predominantly expressed in gametocytes but largely down-regulated in unsporulated oocysts, confirmed the quality of gametocyte cDNA and served as a gametocyte-specific positive control, establishing the lack of gametocytes in merozoite and oocyst samples. The amplification of the *tfp250* gene, specifically expressed in the asexual stages [19], indicated contaminating merozoite cDNA in the gametocyte cDNA sample, as anticipated, at the 134 h time point. Furthermore, amplification of a chicken host-specific lysozyme gene indicated host cDNA was present in both merozoite and gametocyte preparations, also as anticipated.

After optimisation of parasite lifecycle stage cDNA samples, primer pairs were designed to generate PCR products from exons of less than 1 kb in size, where possible. PCRs were performed at optimal annealing temperatures specific for the individual primer pairs and annealing times optimal for predicted cDNA sized products. PCRs were performed at least twice (and normally three times) for each gene product, by different researchers each time. In the case of failed PCRs, primer pairs were redesigned and retested. Results of PCR on the different lifecycle stages of *E. tenella* indicated that 40 of the 45 protease genes could be amplified from parasite cDNA (Table 2). The five PCRs that failed to amplify a product from cDNA were for three of the eight ubiquitinyl hydrolases, the single OTU protease and one of the six subtilisins. However, it was possible to amplify PCR products from gDNA for all five of these proteases that, when sequenced, confirmed primer specificity (data not shown). The failure to amplify a product from cDNA for these genes may be due to genome annotation problems; possibly the sequence targeted by our primers is not transcribed or falls in unpredicted intronic regions. Alternatively, a low abundance of these transcripts may have contributed to the failure to detect cDNA amplification products. Further work will be required to characterize these genes. All other PCR products from cDNA from the four *E. tenella* lifecycle stages were directly sequenced to confirm the correct coding sequence. Expected and actual cDNA amplicon sizes and their corresponding sequence accession numbers are shown in Table 2.

**Table 2:** **Expression of protease genes in merozoites, gametocytes, unsporulated oocysts and sporulated oocysts of *Eimeria tenella***

**Protease group**	**cDNA product**	**Stage specific expression**	**Predicted amplicon size**	**Actual amplicon size**	**Genbank accession number**
**Aspartic Proteases**	**M G UO SO**		**(bp)**	**(bp)**	
Eimepsin 1		Oocyst Specific	357	659	AJ293829
Eimepsin 2		Gametocyte Specific	467	467	JX503496
Eimepsin 3		Merozoite Upregulated	578	578	JX503497
**Cysteine proteases**					
Cathepsin B		Downregulated in Sporulated Oocyst	656	656	JN641867.1
Cathepsin L		Downregulated in Gametocytes	330	300	JX503498
Cathepsin C1		Merozoite Upregulated	547	547	JX503499
Cathepsin C2		Gametocyte Upregulated	273	273	JX503500
Cathepsin C3		Gametocyte and Unsporulated Oocyst Upregulated	436	436	JX546580
Calpain		Merozoite Upregulated	831	806	JX503501
Ubiquitinyl hydrolase 1		Merozoite Upregulated	548	548	JX503503
Ubiquitinyl hydrolase 2		Gametocyte Upregulated	395	395	JX503504
Ubiquitinyl hydrolase 3		Merozoite, possibly Gametocyte Upregulated	698	698	JX503505
Ubiquitinyl hydrolase 4		Merozoite Upregulated	499	499	JX503506
Ubiquitinyl hydrolase 5		Gametocyte Upregulated	674	719	JX503507
Ubiquitinyl hydrolase 6		-	546	-	-
Ubiquitinyl hydrolase 7		-	529	-	-
Ubiquitinyl hydrolase 8		-	303	-	-
OTU protease		-	802	-	-
Pyroglutamyl peptidase		Gametocyte Upregulated	735	771	JX503502
**Metalloproteases**					
Aminopeptidase N 1		Downregulated in Merozoite	602	602	JX503508
Aminopeptidase N 2		Gametocyte Upregulated	636	636	JX503509
ATP-dependant Zn protease 1		Downregulated in Sporulated Oocyst	604	604	XM_001238661.1
ATP-dependant Zn protease 2		Merozoite Upregulated	389	389	JX503516
ATP-dependant Zn protease 3		Downregulated in Sporulated Oocyst	567	1045	JX503517
CAAX prenyl protease		Merozoite Upregulated	368	368	JX503518
Insulysin 1		Merozoite Upregulated	294	294	JX503510
Insulysin 2		Merozoite Upregulated	416	416	JX503511
Insulysin 3		Oocyst Specific	402	697	JX503512
Insulysin 4		Gametocyte Upregulated	367	367	JX503513
Insulysin 5		Merozoite Upregulated	202	202	*
Leucyl aminopeptidase		Merozoite, possibly Gametocyte Upregulated	804	859	JX503514
O-sialoglycoprotease		Merozoite Upregulated	412	412	JX503515
S2P-like protease		Gametocyte Specific	616	1027	JX503519
**Serine proteases**					
Trypsin 1		Gametocyte Specific	613	613	JX503520
Trypsin 2		Gametocyte Upregulated	721	721	JX503521
Trypsin 3		Merozoite Upregulated	654	849	JN987371.1
Subtilisin 1		Gametocyte Specific	260	260	XM_001238722.1
Subtilisin 2		Gametocyte Specific	690	690	XM_001238654.1
Subtilisin 3		Merozoite Upregulated	1171	1084	JX503522
Subtilisin 4		Gametocyte and Unsporulated Oocyst Upregulated	566	566	JX503523
Subtilisin 5		Gametocyte Specific	360	414	JX503524
Subtilisin 6		-	365	-	-
Prolyl endopeptidase		Downregulated in Sporulated Oocyst	534	534	JX503526
Clp protease		Merozoite Upregulated	413	241	JX503525
Rhomboid protease 1		Merozoite Upregulated	554	554	JN987619.1

*M* Merozoites, *G* Gametocytes, *UO* Unsporulated Oocysts, *SO* Sporulated Oocysts.

* = short sequence not accepted for submission.

The majority of the protease genes were expressed in more than one of the four parasite stages investigated (Table 2). However, stage-specific up- or downregulation of protease gene expression was evident. Thus, taking into account that merozoite cDNA contaminates the gametocyte samples, it is safe to conclude that there were a large number (at least 15, probably 17) of protease genes whose expression was upregulated in merozoites including eimepsin 3, cathepsin C1, calpain, several of the ubiquitinyl hydrolases, an ATP-dependent Zn protease, the CAAX prenyl protease, three of the five insulysins, the leucyl aminopeptidase, the O-sialoglycoprotease, one of the trypsins, a subtilisin, the Clp protease and rhomboid protease 1. Aminopeptidase N1 appeared to be downregulated specifically in merozoites. Gametocyte-specific or gametocyte-upregulated proteases were also common, with thirteen in all, also distributed across the four groups of proteases, including eimepsin 2, cathepsin C2, ubiquitinyl hydrolase 2 and 5, the pyroglutamyl peptidase, aminopeptidase N2, insulysin 4, the S2P-like metalloprotease, two trypsin-like proteases and three of the subtilisins. Additionally, two other proteases were upregulated or specific for the sexual phase of the lifecycle (i.e., gametocytes and unsporulated oocysts), namely, cathepsin C3 and subtilisin 4. Cathepsin L appeared to be downregulated specifically in gametocytes. Only two protease genes, a pepsin-like protease with high homology to eimepsin (eimepsin 1) and an insulysin, were switched on exclusively in oocyst lifecycle stages. In contrast, numerous protease genes appeared to be downregulated in sporulated oocysts (Table 2).

### Protease processing of GAM56

**Figure 2:**
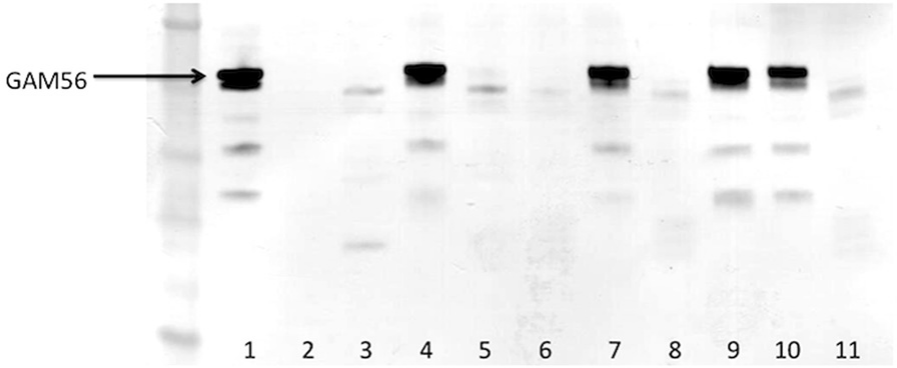
**The effect of protease inhibitors on degradation of GAM56 in *Eimeria tenella *gametocytes.** A sample of purified *E. tenella* gametocytes was lysed and equal volumes of lysate added to a range of protease inhibitors (see Table 3 for details on concentrations and specificity) or PBS. A zero time point sample was taken immediately. Other samples were incubated for 24 h at 37°C, after which the assay was halted by addition to Laemmli buffer and the samples subjected to SDS-PAGE and immunoblotting as described previously [20,21] to assess the disappearance of GAM56. Lane 1, time = zero sample; Lane 2, pepstatin A; Lane 3, E64; Lane 4, aprotonin; Lane 5, AEBSF; Lane 6, bestatin; Lane 7, leupeptin; Lane 8, phosphoramidon; Lane 9, EDTA; Lane 10, a cocktail of pepstatin A, E64, aprotonin, AEBSF, bestatin, leupeptin, phosphoramidon and EDTA; Lane 11, control sample containing no protease inhibitors.

Gametocytes from *E. tenella-*infected caeca were lysed and immediately incubated with or without protease inhibitors for various lengths of time, and the native GAM56 protein analysed by SDS-PAGE and western blotting with anti-GAM56 antibodies, as described previously [20,21], to track the disappearance of the protein to determine whether any inhibitors could prevent the degradation observed in the presence of native gametocyte proteases. The precise epitopes recognised by the anti-GAM56 polyclonal antibodies are not known for *E. tenella* though there is some evidence, from work with *E. maxima*[21], that they are located in the conserved amino-terminus of the protein. The anti-GAM56 antibodies are, thus, very useful for sensitive and specific tracking of the degradation of GAM56. No disappearance of GAM56 was apparent after 2, 4, 6, 8, 10, 12 or 16 h (data not shown) but was obvious at 24h (Figure 2). The 24 h assay was therefore repeated three times with a comprehensive range of protease inhibitors (Table 3) targeting the four protease families identified in the genome. The aspartyl protease inhibitor, pepstatin A, had no effect on GAM56 disappearance (Figure 2). None of three cysteine protease inhibitors investigated, Z-Phe-Ala-diazomethylketone (data not shown), N-ethylmalemide (data not shown) or E64 (Figure 2) inhibited GAM56 disappearance. The serine/cysteine protease inhibitor, chymostatin (data not shown) and leupeptin (Figure 2), inhibited GAM56 disappearance but another inhibitor with the same specificity, antipain, did not (data not shown). The serine protease specific inhibitors, benzamidine HCL (data not shown), soybean trypsin inhibitor (data not shown) and aprotinin (Figure 2) all inhibited the disappearance of GAM56 but AEBSF did not (Figure 2). The metal chelating agent, EDTA, also inhibited the disappearance of GAM56 but more specific metalloprotease inhibitors, bestatin and phosphoramidon, did not (Figure 2).

**Table 3:** **Protease inhibitors used in the *Eimeria tenella *** **GAM56 processing assay**

**Inhibitor**	**Inhibitor target**	**Final concentration**	**Company**
Pepstatin A	Aspartyl	1 μm	Sigma-Aldrich
N-ethylmalemide	Cysteine	1 mM	Sigma-Aldrich
E-64	Cysteine	10 μM	MP Biomedicals
Z-Phe-Ala-diazomethylketone	Cysteine	2 μM	Bachem, UK
Antipain	Ser + Cys	100 μM	MP Biomedicals
Chymostatin	Ser + Cys	200 μg/ml	Sigma-Aldrich
Leupeptin	Ser + Cys	100 μM	Sigma-Aldrich
AEBSF	Serine	1 mM	Sigma-Aldrich
Aprotinin	Serine	0.002 TIU	MP Biomedicals
Benzamidine HCl	Serine	4 mM	Sigma-Aldrich
Soybean Trypsin inhibitor	Serine	100 μg/ml	Sigma-Aldrich
Bestatin	Metallo (amino)	40 μM	Sigma-Aldrich
Phosphoramidon	Metallo (endo)	10 μM	MP Biomedicals
EDTA di-sodium salt	Metallo	5 mM	Sigma-Aldrich

## Discussion

Mining of the *E. tenella* genome database has revealed over 40 protease transcripts distributed over 13 clans and 18 families of aspartic, cysteine, metallo and serine proteases. Such diversity of proteases is not unusual, indeed it may be an underestimate of the true number of protease genes in this parasite since other apicomplexan parasites are known to possess substantially more protease genes (Table 4); thus, for example, there are at least 70 in *Cryposporidium parvum*, more than 80 in *P. falciparum* and over 90 in *T. gondii,* though other apicomplexan parasites possess similar numbers of protease genes as *E. tenella. Eimeria tenella* also has lower numbers of protease genes than protozoan parasites like *Leishmania, Trypanosoma* and *Trichomonas* (though the latter is known to have an unusually expanded genome in general [22] and, apparently, in C1 and C19 cysteine proteases and M8 metalloproteases, in particular; Table 4). But, again, *E. tenella* has a broadly similar total number of protease genes to *Entamoeba dispar* and *Giardia intestinalis*, which are also intestinal parasites. However, the fact that our dataset for *E. tenella* lacks protease genes for several families, across all four types of proteases, that are represented in all other Apicomplexa and most other protozoan parasites, including A28, A22, C12, C85, C86, C13, C14, C50, C48, M24, M18, M67, S9, S26 and S16, provides reason to believe that some *E. tenella* protease genes remain unannotated.

**Table 4:** **Distribution of protease clans and families in *Eimeria tenella*, its host and other protozoan parasites**

**Clan**	**Family**	***G.g***	***E.t***	***T.g***	***C.p***	***C.h***	***C.m***	***P.f***	***P.v***	***P.b***	***P.c***	***P.y***	***T.a***	***T.p***	***E.d***	***L.b***	***L.i***	***L.m***	***T.b***	***T.c***	***T.v***	***G.i***
AA	A1	11	3	8	4	3	6	11	6	5	3	9	4	3							1	
	A2	3																				
	A28	1		1	2	1	1	1	1	1	1	2	1		1	1	1	1	1	1	2	6
AD	A22	6		1	1	1	1	1	1	1		1			4	2	2	2	2	2	4	2
CA	C1	12	5	4	5	4	4	10	9	8	4	8	12	10	15	3	4	3	2	7	42	21
	C2	12	1	2	1		1	2	1	2	2	1	1		1	2	2	2	1	2	4	
	C12	4		2	2	2	1	2	1			2			2	2	1	2	2	2	1	
	C19	30	8	1	7	3	2	7	1	2	3	4	5	1	1	3	1	9	8	3	68	4
	C51															1	1	2	3	2		
	C54	3			1		1	1							4	2	2	2	2	2	5	
	C64	4																				
	C65			1												1	1	1	1	1		
	C66																				1	
	C67	2																				
	C78	1		1	1		1						1	1		1	1	1	1	1		
	C85	2		1	1	1	2	1	1			1										
	C86	2		1	1	1	1	1	1	1		1										
	C88	1	1	1											1	1	1	1	1	1	1	
CD	C11			1	1		1															
	C13	2		1	2	1	1	1	1			1	1	1	1	1	1	1	1	1	9	2
	C14	12		2	1	1	1	1		1	1					1	1	1	2	2	6	
	C50			1	1	1	1								1		1	1	1	1	1	1
CE	C48	6		3	2	1	1	1	1	1	2	2	2	2	1				1	1	6	
CF	C15	2	1	1															1	1	1	
CO	C40																				6	
M-	M49														1	1	1	1			1	2
	M76	1		3				1	1	1	1	1				1	1	1	1	2		
	M79							1		1	1		1						1	2	1	1
MA	M1	10	2	3	1	1	1	1		1	1	1	1	1	1	2	2	4	2	3	3	
	M2	3																				
	M3	3		3				2	2	2	2	3		1	2	3	3	4	2	3	1	
	M8	1													1	24	8	4	9	76	42	
	M10	16																				
	M12	35																				
	M13	8		1																		
	M32															2	3	4	1	2		
	M41	1	3	3	1		1	3	3	3	3	4	3	3		1	5	5	5	5	1	
	M43	2																				
	M48	1	1	1	1	1	1						1	1	1	1	1	1	1	1	4	
	M54	1			1	1	1															
	M80							1	1	1						1	1	1	1	1		
MC	M14	14			1	1	1	1								2		2	3	3	3	4
ME	M16	12	5	7	8	4	4	4	3	4	1	4	5	4	2	2	3	3	3	3	7	3
MF	M17	3	1	1	1	1	1	1	1	1	3	2	1	1		3	3	3	3	3		
MG	M24	6		3	2	3	2	5	4	4	3	5	4	4	3	3	3	4	4	4	8	3
MH	M18	1		1	1	1	1	1	1	1	1	1	1	1	1	1	1	1	1	1	5	
	M20	4		1			1								6	3	4	7	4	8	10	3
	M28	10																1			1	
MJ	M19	1																				
	M38	4																				
MK	M22	2	1	1	1	1	1	2	1	1	1	2	2	1	1	1	1	1	1	1	1	2
MM	M50	1	1					1														
MP	M67	7		2	2	2	1	1	1	1	1	1	2	2	2	2	2	3	1	3	4	2
PA	S1	66	3	4				1	2				1									
SB	S8	10	6	11	1		1	3	3	2	2	2						1	2	2	11	
SC	S9	15		4	10	3	2	3	1	1		2	2	1	2	5	4	7	8	8	8	5
	S10	2														1	1	1	3	1		
	S15	1		1		1												1		1		
	S28	1		1											2					1	8	2
	S33	3	1	2	3	1		6	1	1	4	1			1	2	1	2	1	2	13	7
SE	S12																				1	
SF	S26	4		1	1		1	1	1	1	1		2	2	1		2	2	2	2	1	3
SJ	S16	2		1			1	1	1	1		1	1	1								
SK	S14		1					1	1	1	1	1	2	1								
	S41	1																				
SP	S59	1		1	1		1															
SR	S60	1																				
ST	S54	6	1	6	3	2	3	7	2	4	2	5	1	1	1						2	
Clan	Family	***G.g***	***E.t***	***T.g***	***C.p***	***C.h***	***C.m***	***P.f***	***P.v***	***P.b***	***P.c***	***P.y***	***T.a***	***T.p***	***E.d***	***L.b***	***L.i***	***L.m***	***T.b***	***T.c***	***T.v***	***G.i***

The distribution of families within clans of aspartic (AA, AD), cysteine (CA, CD, CE, CF, CO), metallo (M-, MA, MC, ME, MF, MG, MH, MJ, MK, MM, MP) and serine (PA, SB, SC, SE, SF, SJ, SK, SP, SR, ST) proteases of *Eimeria tenella* is compared with its host (*Gallus gallus,* the chicken) and other protozoan parasites from the MEROPs database of peptidases in whole genome sequences (merops.sanger.ac.uk/cgi-bin/compgen_index?type=P; [34]). Sequences for non-peptidase homologs are not included in the table. *G.g = Gallus gallus; E.t = Eimeria tenella; T.g = Toxoplasma gondii; C.P. = Cryptosporidium parvum; C.h = Cryptosporidium hominis; C.m = Cryptosporidium muris; P.f = Plasmodium falciparum; P.v = Plasmodium vivax; P.b = Plasmodium berghei; P.c = Plasmodium chabaudi; P.y = Plasmodium yoelii; T.a = Theileria parva; P.a = Theileria annulata; E.d = Entamoeba dispar; L.b = Leishmania braziliensis; L.i = Leishmania infantum; L.m = Leishmania major; T. b = Trypanosoma brucei; T.c = Trypanosoma cruzi; T. v = Trichomona vaginalis; G.i = Giardia intestinalis.*

The apparent stage-specific regulation of protease genes in *E. tenella* is striking and intriguing. Most investigations of parasitic protozoan proteases have focused on the asexual stages of the apicomplexan parasites, *T. gondii* and *P. falciparum,* establishing crucial roles for proteases in host cell invasion, remodelling and egress by the asexual stages of these parasites [1]. Our finding that expression of up to 17 of 40 protease genes examined in *E. tenella* is upregulated in merozoites further underscores the importance of proteases in the biology of the asexual stages of apicomplexan parasites. Not surprisingly, therefore, an eimepsin, several cathepsins, a calpain, a trypsin-like protease, subtilisins, Clp and a rhomboid protease are upregulated in the asexual stages of *E. tenella* (Table 2). Likewise, eimepsin1 and insulysin 3 are expressed specifically in oocysts and may play an important role in the first steps of the parasite lifecycle, such as host cell invasion; they are, therefore, worthy of further research. The downregulation of several proteases (including cathepsin B, ATP-depenedent ZN proteases 1 and 3, and a prolyl endopeptidase) in sporulated oocysts may be, in part, attributed to the dormancy of this lifecycle stage, yet still warrants further investigation.

Perhaps the most significant finding of our stage-specific expression study was the relatively large number of protease genes whose expression is upregulated specifically in the gametocytes stage – a total of at least 13 genes, including six that are only expressed in gametocyte (Table 2). This observation becomes even more intriguing when examined in the context of the distribution of different families of proteases across parasitic protozoa (Table 4). Four classes of proteases stand out amongst the protozoa because they are only found, or are “over-represented” in the two Coccidian parasites, *E. tenella* and *T. gondii* – families C15, M50, S1 and S8. *Eimeria tenella* contains a total of eleven protease genes distributed unevenly across these families, with only one in C15 and M50 and three and six in the serine protease families, S1 and S8, respectively. But, even more significantly, all but three of these unique protease genes are upregulated or confined in expression to the gametocyte stage of the parasite. Thus, expression of a pyroglutamyl peptidase, a trypsin-like protease and subtilisin 4 is upregulated in gametocytes whilst expression of an SP2-like protease, a trypsin 1-like protease and three subtilisins is entirely gametocyte specific.

One of the defining features of the Coccidia is the possession of a hard-walled oocyst that originates from specialized organelles (wall forming bodies) in macrogametocytes. It is hypothesized [16] that degradation of two proteins found in the wall forming bodies of macrogametocytes of *Eimeria,* namely GAM56 and GAM82, is integral to oocyst wall formation; tyrosine-rich peptides formed by the degradation of these two proteins are believed to be subsequently cross-linked via dityrosine bonds [23], giving the oocyst wall its renowned strength and resistance to environmental and chemical insults [24]. To test this hypothesis, we designed an assay to follow the degradation of GAM56 in freshly harvested gametocytes (Figure 2). This assay has certain inherent limitations: first, it relies on sensitive antibodies for detection of specific degradation of GAM56 and, unfortunately, the lack of suitable antibodies for detection of GAM82 in *E. tenella*[21] meant that we were unable to run confirmatory experiments with this protein; and, second, the only controls possible are a zero time point and a cocktail of protease inhibitors designed to prevent all proteolytic activity. These limitations require us to be cautious in our interpretations; none-the-less, the inhibition of degradation of native GAM56 by a very specific group of protease inhibitors reveals that this function may be carried out by subtilisin-like proteases. Thus, degradation of GAM56 was inhibited by the serine/cysteine protease inhibitors, chymostatin and leupeptin, and the serine protease specific inhibitors, benzamidine HCL, soybean trypsin inhibitor and aprotinin but not by AEBSF (Figure 2). Intriguingly, the metal chelating agent, EDTA, also inhibited degradation of GAM56. This profile indicates that serine proteases are critical for degradation of GAM56 but it seems to rule out participation of rhomboid proteases, which are unaffected by EDTA, aprotonin, leupeptin and chymostatin [25]. Trypsin-like proteases can, perhaps, not be completely ruled out of this process but the inhibitory profile, particularly the lack of inhibition by AEBSF coupled with the inhibitory effect of EDTA, points to a subtilisin or subtilisins as *P. falciparum* subtilisin 1 is inhibited in exactly the same fashion [26].

Subtilisins are further implicated in the formation of the oocyst wall of *Eimeria* through analogy with their known role in the formation of the cuticle of nematodes. Thus, the assembly of collagens to form the cuticle involves a number of molecular events that strikingly resemble our model of oocyst wall formation pathways: first, collagens are the result of degradation of proproteins by a subtilisin-like protease [27-30]; and, second, these collagens are subsequently bonded together by di- and tri-tyrosine crosslinks [31]. A failure in either of these steps, results in a malformed cuticle and parasite death [31]. Subtilisins are currently being further investigated as potential candidates in the catalytic cleavage of the oocyst wall precursor proteins.

## Conclusion

*Eimeria tenella* possesses a large number of genes coding for proteolytic enzymes, which display a remarkable pattern of stage specific expression. As in other apicomplexan parasites such as *P. falciparum* and *T. gondii,* expression of many of these genes is upregulated in the asexual, invasive stages, possibly indicating important roles in host cell invasion, remodelling and egress. However, expression of almost one-third of the protease genes identified in the *E. tenella* genome is upregulated or confined to the sexual gametocyte stage of this parasite’s lifecycle; some of these appear to be unique to Coccidia and may play key roles in the formation of the resilient oocyst wall, a defining feature of this group of important parasites.

## Methods

### Data-base mining

*Eimeria tenella* genome sequences and gene models were downloaded from GeneDB (http://www.genedb.org/Homepage/Etenella). The genome of *E. tenella* (Houghton strain) was produced by the Parasite Genomics Group at the Wellcome Trust Sanger Institute (http://www.sanger.ac.uk/research/projects/parasitegenomics/) and has been provided prepublication. The *E. tenella* genome database was searched for genes predicted to code for proteins with peptidase activity. All auto-annotated peptidase genes identified were manually curated by performing BLAST analysis against apicomplexan genome sequence databases and against various protein databases [32] such as the protein data bank (PDB), Swiss-Prot and non-redundant (NR) protein sequence databases. In addition, signature protein motifs for the protein sequence of each gene were identified through Pfam (http://pfam.sanger.ac.uk/search; [33]), InterproScan (http://www.ebi.ac.uk/Tools/pfa/iprscan/) and the MEROPS databases (http://merops.sanger.ac.uk/; [34]). Further gene sequence manipulations, such as translation into amino acid sequences and ClustalW alignments, were performed using the DNASTAR Lasergene™ 9 Core Suite. After the bioinformatic information was collated, genes were assigned a five-tiered level of confidence for gene function using an Evidence Rating (ER) system giving an overall score of ER1-5, where ER1 indicates extremely reliable experimental data to support function and ER5 indicates no evidence for gene function [17].

### Animals and parasites

One day old chicks (Australorp; Barter and Sons Hatchery, Luddenham, Australia) were housed at the Ernst Facility Animal House (University of Technology, Sydney), under heat lamps for the first 2 weeks of their life and, thereafter, at 21°C with a 12 hour light/dark cycle with free access to food and water. Chickens were infected orally at 4.5 weeks of age with 2.5 × 10^3^ sporulated oocysts of *Eimeria tenella* (Houghton strain originally provided by Janene Bumstead, Institute for Animal Health, Compton, UK). Fresh *E. tenella* oocysts were harvested 7 days post infection from the caeca following protocols published previously [35]. Sporulation of oocysts was carried out at 28°C for 72–120 hours using a low-pressure aquarium pump to aerate the suspension. Sporulated oocysts were then treated with 2.8 M NaCl and 2% sodium hypochlorite (Milton solution) and stored in 2% potassium dichromate at 4°C until required. Unsporulated oocysts were also treated with Milton solution and stored at −80°C. Merozoites (112 h p.i.) and gametocytes (134 h p.i.) were isolated from infected chicken caecae following techniques published previously [36,37]. Aliquots of parasites were either frozen at −80°C as pellets or were stored in TRIzol® reagent (Invitrogen) at −80°C for further use in RNA purification.

### RNA purification, cDNA synthesis and cDNA standardisation

To isolate total RNA, purified merozoites (1 × 10^7^) and gametocytes (1 × 10^6^) were resuspended in 1 ml TRIzol® Reagent and homogenized by pipetting. Unsporulated oocysts (2 × 10^5^) and sporulated oocysts (5 × 10^5^) were resuspended in 1 ml TRIzol® Reagent and one volume of glass beads were added to the sample, which were then vortexed for 1 min intervals until disruption of oocyst was confirmed by bright field microscopy. All TRIzol® treated samples were left at room temperature for 10 min and total RNA isolated by chloroform extraction and isopropanol precipitation. RNA was quantified using a NanoDrop™ ND-1000 Spectrophotometer and cDNA was synthesized using SuperScript™ III Reverse Transcriptase (Invitrogen) according to manufacturer’s instructions.

Parasite cDNA samples were standardized by relative quantification of an *E. tenella* β-actin PCR product. β-actin forward primer E0043 (5^′^ ggaattcgttggccgcccaagaatcc 3^′^) and reverse primer E0044 (5^′^ gctctagattagctcggcccagactcatc 3^′^) were used to generate the 1020 bp β-actin cDNA PCR product. Each PCR reaction contained 50 ng of parasite stage specific cDNA, 0.2 μM forward primer, 0.2 μM reverse primer, 1 × AccuPrime™ reaction mix, and AccuPrime™ *Pfx* DNA polymerase (Invitrogen). The PCR reaction was carried out as follows: initial denaturation 95°C for 3 min; 95°C for 30 s; 61°C for 1 min; 68°C for 1.5 min, for 25 cycles with a final extension at 68°C for 10 min. All products were electrophoresed on a 1% agarose gel and visualized using Gel Red™ (Biotium). The net intensity of each band was determined using the Kodak EDAS 290 Electrophoresis Documentation and Analysis System and serial dilutions performed until relative intensity of PCR products were equal.

In addition, three control genes were amplified to determine the purity of parasite lifecycle stages. The GAM56 gene was used as a gametocyte specific gene. GAM56 forward primer E0030 (5^′^ catatggtggagaacacggtgcac 3^′^) and reverse primer E0031 (5^′^ ctcgagttagtaccagctggaggagta 3^′^) were designed to amplify a 906 bp gametocyte cDNA product at an annealing temperature of 61°C. The EtTFP250 gene, a homolog of an *E. maxima* gene encoding a microneme protein, was used as an asexual stage control. The EtTFP250 forward primer Et250F (5^′^ gcaaggacgttgacgagtgtg 3^′^) and Et250RV1 (5^′^ gttctctccgcaatcgtcagc 3^′^) were designed to amplify an 805 bp cDNA product, at an annealing temperature of 60°C. The chicken lysozyme gene was used to determine relative quantities of contaminating host cDNA. The forward primer RW3F (5^′^ acaaagggaaaacgttcacgattggc 3^′^) and reverse primer RW4R (5^′^ tgcgttgttcacaccctgcatatgcc 3^′^) were designed to amplify a 280 bp host cDNA product at an annealing temperature of 60°C.

### Semi-quantitative PCR

The predicted coding regions of each protease gene were examined for potential primer sites within 1 kb of each other where possible. Primers were designed as detailed in Table 5.

**Table 5:** **Primers for *Eimeria tenella *** **protease genes for the amplified coding sequences**

**Protease gene**	**Primer**	**Primer sequence 5**^**′**^** to 3**^**′**^
**Aspartic Proteases**		
Eimepsin 1	F	ATC ACC ACA CCA CCA TGG G
	R	CAA GAT TCG AGC AGT TCT CAG CA
Eimepsin 2	F	AGC AAA CAG CTG CAG ATG TTC C
	R	CGA AAA TAG GTC TAG GGG CCC
Eimepsin 3	F	AGA AGT CCT TTC CTC CGT CAC G
	R	GGC GAA GTG TAT TGA AAG CTC G
**Cysteine Proteases**		
Cathepsin B	F	TGA CGT GGG AAG CAG AAG TGT C
	R	ACT GTA TGC ACT CGT GGC GAA A
Cathepsin L	F	CAA CCA ACA AGG TCA CTC TTA C
	R	CCC TGG AGG GCC CCC GTG CTC G
Cathepsin C1	F	AAC GGA AGT GGA GAA GAC AGC A
	R	CGG GGT CAA TGA AGG AAG TTT G
Cathepsin C2	F	GGC TTT GAA GTT TGG CAG CG
	R	TGC ATC TGA GCA GCA GAA GAG
Cathepsin C3	F	CGC TCA GGA GTA CAA CTA CGT GGG TGG
	R	GCT GCT GCA CAA GCA GCG CTG CTC TGC C
Calpain	F	CGA CTG CTC CTT CCT CTC
	R	GCA TCC TTT AGC TGC TGG
Ubiquitinyl hydrolase 1	F	CTG GCT CTT GAA TGT GCT GCA T
	R	CTT CCA TTT CGT GCC ATT CG
Ubiquitinyl hydrolase 2	F	TTG GAC TTC AGC CCG AGG AG
	R	CCG CTC GTC AAA TCC CAA AG
Ubiquitinyl hydrolase 3	F	AGG TGG TGC CCC TCG ACA TA
	R	TTC TGC TGC CGT GCT CTT TG
Ubiquitinyl hydrolase 4	F	GTG AAT GTG GGC AGC ACC AG
	R	GCT TTC CTG CAA GGC GAA GA
Ubiquitinyl hydrolase 5	F	TCT CTT CCA GGG GCA GTA CAG G
	R	GCT GCT CGT AGG CCA TAT TTG AAC
Ubiquitinyl hydrolase 6	F	GGT GGC CAG CAT AGA CGA GAG T
	R	CCG TAG GTT TGC TGC GAT CTC T
Ubiquitinyl hydrolase 7	F	CGA CAG TCC CGT TGT TGC TG
	R	AGC AAG CCG GGG AAA GAG AC
Ubiquitinyl hydrolase 8	F	TCC TGG AGG CCT TGA GCA GT
	R	TGC TGC TCA CTG GGA TGT GG
OTU protease	F	ATG GAC GTA GCA ATC CGT TAC
	R	GTT GCT GCA CAT AGT CCC AAG
Pyroglutamyl peptidase	F	ATG GGA CAA CCT ACA GGC GAG
	R	GAG GAA GTT ACA AAC GAA GCA GC
**Metallo Proteases**		
Aminopeptidase N 1	F	CAA GCA GTA CAC TCC AGC AAC TC
	R	GAA GCG TCA GGT GTT CGT ATT CC
Aminopeptidase N 2	F	GAT ACA TCG CAA GGA CTA CAG C
	R	CAT TGT GCG GAA GGA GTC TG
ATP-dependant Zn protease 1	F	CTT CGA CCA GCT GAA GAT CCT G
	R	TGT CTC CGC GCT AAT GCT G
ATP-dependant Zn protease 2	F	CTC AAT GGT GGA TTT CAG CAA G
	R	CAT CAT TCC CTG GCT TGC G
ATP-dependant Zn protease 3	F	GTC AAG AAA GCA GAT TGT CAG G
	R	CTC GAC AGC CAT CTC AAA GTC
CAAX prenyl protease	F	GAA TGT CCC AGG AGA GCT ATG C
	R	GTT CAC AGC GCA CAG AAT TGG
Insulysin 1	F	CAC TTC CTG GAG CAC ATG G
	R	GTG CAG CCT GTC GAA GAT G
Insulysin 2	F	CCG CGG TAG CCT CTCA GC
	R	CTC CGC TGT TGC TCA CAT CTT C
Insulysin 3	F	GAT TGA CGC CGA GCA CCA G
	R	GCT AGG CGA GCT GCT TCA TCC
Insulysin 4	F	GTC GTG CAG AGA GGC TGG
	R	GAG GTA GCT GAG TCG GAG GG
Insulysin 5	F	ATG ATA TTT CTG AAG TCT GC
	R	GAG GAT TCA AAA GAT GGT C
Leucine aminopeptidase	F	CAA GAT CTA GTT GTT TGA GGA GCC C
	R	GCT GTT CCA CTT CAC TCG TCT TC
O-sialoglycoprotease	F	GCA AAT GTG ACG CAG CTG ATT CG
	R	CGA ACT TGA GGG CCA TTT GCT C
S2P-like protease	F	GCC CTC GTA TAA CAG CAA C
	R	GAA CGA CTA ACT TCC TCT TGC
**Serine Proteases**		
Trypsin 1	F	GTA GGC AAC GGA ACT CCA GC
	R	CGA GGA CTA CAA CTG TCT CC
Trypsin 2	F	ATG GAA GCG TCT GGT TGG GAC
	R	GCT TTT GCT GCA TGC ACT C
Trypsin 3	F	GAC AAA CAA GTT CAA CGA GCA CTG
	R	GCT TCC CTC TCC GCA GAA TTG
Subtilisin 1	F	TAA TTA CCT CCA TCC CGA ACT G
	R	CCA GAA TCT TCA GCG CCA TCA C
Subtilisin 2	F	GCA GCA GCA AAT GTT GAA GAC CC
	R	ATA AGT GCT GCT GCC AAC CAC C
Subtilisin 3	F	AGA GCT TTT GTC CGT GGT GGA G
	R	AAA GAC CCC GAA AAC CAA TGC T
Subtilisin 4	F	CCT TTG TGG CGT GTT CGT GAG
	R	CCA GCA GAA GCA GTA CCG TGG CC
Subtilisin 5	F	TTG AAG CCG ACA GGA CGT GG
	R	CCG CGT AGT CAA GAG CAG GGA T
Subtilisin 6	F	AGC GGC TGC GAC TTG AAC C
	R	CCG TAG CCG CCG TAG GAG TT
Prolyl endopeptidase	F	ACA GCC AGG CAC ATC AAT GGT
	R	GCC AAA CCC AAG CCC AGA TAG
Clp protease	F	GCT GCA CTT CCA GAA GCG G
	R	CCT CCG CAG AGA AGA CTT TGC
Rhomboid protease 1	F	GGT TGT CCG CAC GTT GGC AG
	R	CGA AGA TAA CAG GCA CGC AAG ATG

PCRs were conducted on cDNA samples from *E. tenella* merozoites, gametocytes, unsporulated and sporulated oocysts. PCR were optimized to produce cDNA sized products. Negative controls of no DNA template and host cDNA were run alongside a positive genomic DNA control. When genomic DNA products were not amplified, a repeat PCR was performed at longer annealing times to produce the often much larger genomic DNA product. A typical PCR was as follows: 1μL of standardized cDNA sample, 0.2 μM forward primer, 0.2 μM reverse primer, 1 × AccuPrime™ reaction mix, and AccuPrime™ *Pfx* DNA polymerase (Invitrogen). Cycling conditions typically involved an initial denaturation at 95°C for 3 min, followed by 25 cycles of denaturation 95°C for 30 s; annealing at Tm-5 for 1 min; extension at 68°C for 1.5 min. When products were to be sequenced, a final extension at 68°C for 10 min was performed at the end of the PCR reaction. PCRs were performed at least twice and, generally, three times for each gene product by a different researcher each time.

All amplified products were gel purified using a QIAquick® Gel Extraction Kit (QIAGEN) according to the manufacturer’s instructions and sequenced (Australian Genome Research Facility, Queensland). When cDNA products were amplified from different parasite stages, these were pooled and used in sequencing reactions. When cDNA products were not obtained, additional primers were designed and used. If a cDNA product was still unable to be amplified with the second primer pair, genomic DNA products were sequenced to confirm primer specificity. Sequences were analysed using DNASTAR Lasergene™ 9 Core suite.

### GAM56 processing assay

A frozen sample of purified *E. tenella* gametocytes (1 × 10^6^ cells in 100 μL) was resuspended in PBS (145 mM NaCl, 7.5 mM Na_2_HPO_4_, 2.5 mM NaH_2_PO_4_.2H_2_O, pH 7.4) to a final volume of 500 μL. Glass beads (250 μL of 710-1180 μm, Sigma) were added to the suspension and vortexed at full speed for three 1 min pulses with a 1 min pause on ice between each pulse. After three vortex cycles, the sample was centrifuged and the lysate transferred to a clean tube. Equal aliquots of the gametocyte extract (18 μL) were immediately added to either 2 μL of 10× protease inhibitor (Table 3) or PBS. A zero time sample was taken from the PBS control and immediately added to Laemmli sample buffer [38] and frozen. The assay tubes were incubated at 37°C for 2, 4, 6, 8, 10, 12, 16 or 24 h, after which Laemmli sample buffer was added and samples stored at −20°C for further assessment.

SDS-PAGE and immunoblotting were carried out as described previously [20,21]. Briefly, gametocyte assay samples, resuspended in Laemmli sample buffer (5 μL), were boiled for 5 min at 100°C prior to SDS-PAGE on a NuPAGE® Novex 4-12% Bis-Tris Gel (Invitrogen). SeeBlue® Plus2 Pre-Stained Standards (Invitrogen) were used as a marker. Proteins separated by SDS-PAGE were transferred electrophoretically (100 V, 1 hour, 4°C) to Immobilon-P membrane (Millipore) in transfer buffer (25 mM Tris–HCl, 192 mM glycine, 10% methanol). Membranes were rinsed in methanol and water then soaked for 10 min in transfer buffer prior to transfer. Gels were pre-soaked for 15 min in transfer buffer. After transfer, membranes were incubated overnight in blocking solution (5% w/v skim milk powder in PBS) at 4°C and then incubated with primary antibody (1:1000 rabbit anti-EmGAM56) for 2 h at room temperature. Each membrane was washed twice for 5 min and twice for 10 min in 0.05% Tween 20 in PBS then incubated with secondary antibody (1:1000 anti-rabbit IgG conjugated to alkaline phosphatase; Sigma) for 2 h at RT. Membranes were washed as above and bands visualized with SIGMA *FAST*™ BCIP/NBT buffered substrate (Sigma).

## Competing interests

The authors declare they have no competing interests.

## Authors’ contributions

NCS, MK and SIB conceived the study. MK coordinated the study and performed data analysis, along with FB, FMT and NCS. MK, RJI, MR, IS, PAS and RAW carried out the laboratory work. NCS, MK, FMT and FB drafted the manuscript. All authors read and approved the final manuscript.
